# Exploring the Intersection of Chronic Disease, Function, and Social Care in Adult Day Care Clients With Dementia

**DOI:** 10.1093/geroni/igab046.319

**Published:** 2021-12-17

**Authors:** Tina Sadarangani, Jonelle Boafo, Bei Wu, Abraham Brody, Gary Yu

**Affiliations:** 1 New York University Rory Meyers College of Nursing, New York University Rory Meyers College of Nursing, New York, United States; 2 Rory Meyers College of Nursing, New York, New York, United States; 3 New York University, New York, New York, United States; 4 NYU Hartford Institute for Geriatric Nursing, New York, New York, United States; 5 NYU Rory Meyers College of nursing, New York, New York, United States

## Abstract

The interrelationships among dementia, concomitant disease, and social determinants of health are poorly understood and have critical implications for disease course, treatments, and caregiving needs. The aim of this study was to identify patterns of co-occurring chronic conditions among persons with dementia and the relationship of these patterns with clinical characteristics, demographic predictors and functional status. A latent class analysis (LCA) was conducted using data from 53 California adult day centers (n=3,053). Four distinct groups emerged: “dementia only”; “dementia +: > 2; + > 3; + >5 chronic conditions. Having dementia + >5 was associated (p <.001) with greater risk of falls, isolation, medication mismanagement, and reduced likelihood of using an adaptive device. Dementia in day center clients is complicated by clinical conditions, functional decline, and a need for supports that may be lacking. Center staff must be trained and resourced to manage the complex needs of persons with dementia.

